# Machine Learning Radiomics in Computed Tomography for Prediction of Tumor and Nodal Stages in Colorectal Cancer

**DOI:** 10.3390/cancers18030377

**Published:** 2026-01-26

**Authors:** Lara de Souza Moreno, Tony Alexandre Medeiros da Silva, Mayra Veloso Ayrimoraes Soares, João Luiz Azevedo de Carvalho, Fabio Pittella-Silva

**Affiliations:** 1 Laboratory of Molecular Pathology of Cancer, Faculty of Health Sciences, University of Brasília, Brasília 70910-900, DF, Brazil; larasmoreno@gmail.com (L.d.S.M.); mayraveloso@gmail.com (M.V.A.S.); 2 Department of Informatics, Goiano Federal Institute, Ipameri 75780-000, GO, Brazil; tony.medeiros@ifgoiano.edu.br; 3 Department of Electrical Engineering, University of Brasília, Brasília 70910-900, DF, Brazil; joaocarvalho@unb.br

**Keywords:** radiomics, machine learning, colorectal cancer, computed tomography, tumor staging, nodal metastasis

## Abstract

**Background/Objectives**: Accurate preoperative TN staging is essential for guiding surgical and adjuvant treatment decisions in colorectal cancer (CRC), yet conventional imaging still faces limitations in reliably distinguishing early from advanced disease. This study aimed to evaluate whether CT-based radiomics combined with machine learning can noninvasively predict both tumor (T) and nodal (N) stages of CRC, and to identify which feature groups most contribute to each task. **Methods**: Fifty-three patients (55 tumors) with histologically confirmed CRC who underwent preoperative contrast-enhanced CT were retrospectively analyzed. A total of 107 radiomic features were extracted using PyRadiomics version 3.1.0, including shape, first-order, and texture features. Multiple preprocessing strategies—z-score normalization, PCA, and SMOTE—were tested across 11 machine learning classifiers. **Results**: For T staging, logistic regression using shape-based features achieved a mean sensitivity of 0.721, a specificity of 0.68, a balanced accuracy of 0.70, and an AUC of 0.751. For N staging, the AdaBoost model using texture-based features achieved a sensitivity of 0.742, a specificity of 0.622, a balanced accuracy of 0.682, and an AUC of 0.750. Shape features predominantly contributed to T prediction, while texture matrices drove N prediction, reflecting morphological and microstructural correlates of invasiveness and lymphatic dissemination. **Conclusions**: CT-based radiomics can quantitatively capture both morphological and textural patterns of tumor behavior, providing a noninvasive framework for preoperative TN staging in CRC.

## Simple Summary

Colorectal cancer is a common disease in which treatment decisions depend greatly on how deeply the tumor grows and whether it has already spread to nearby lymph nodes. However, standard scans often struggle to clearly show these details before surgery. Radiomics is a new imaging approach that extracts many invisible patterns from routine scans to better understand tumor behavior. In this study, we analyzed computed tomography images from patients with colorectal cancer and used computer-based models to determine whether radiomic patterns could help identify early or advanced tumors and predict lymph node involvement. We found that specific image features related to tumor shape and texture provided valuable information for both tasks. These findings suggest that radiomics could become a useful, noninvasive tool to improve preoperative evaluation and support more personalized treatment planning in the future.

## 1. Introduction

Colorectal cancer (CRC) is the third most frequently diagnosed malignancy and the third leading cause of cancer-related mortality worldwide, accounting for nearly 2 million new cases and 1 million deaths annually [1]. In the United States alone, the American Cancer Society projects approximately 154,270 new CRC cases and 52,900 related deaths for 2025 [2]. Its etiology is multifactorial, encompassing environmental exposures, dietary and lifestyle factors, and hereditary cancer syndromes such as familial adenomatous polyposis and Lynch syndrome [3,4]. Despite improvements in screening and therapy, CRC remains a major public health challenge.

Staging is the cornerstone of CRC management and is based on the TNM classification of the American Joint Committee on Cancer (AJCC) [5]. Accurate preoperative assessment of tumor depth (T stage) and nodal involvement (N stage) is critical, as these parameters directly influence surgical decisions, the need for neoadjuvant or adjuvant therapy, and overall prognosis. T3–T4 tumors demonstrate more aggressive behavior, higher recurrence risk, and greater need for multimodal therapy compared with T1–T2 lesions. Patients with stage III disease commonly benefit from adjuvant chemotherapy [6], whereas for stage II disease, the benefit is confined to those with high-risk features and remains under debate [7]. However, conventional imaging still faces limitations in precisely distinguishing early from advanced disease, increasing the risk of over- or undertreatment.

Radiomics has emerged as a powerful strategy to overcome these challenges. By extracting high-dimensional descriptors of tumor morphology, attenuation, and texture, radiomics enables quantitative and reproducible characterization of tumor biology [8,9,10]. Seminal contributions from Gillies et al. [11], Aerts et al. [12], and Lambin et al. [13] demonstrated that radiomic features can capture imaging phenotypes linked to tumor heterogeneity and clinical outcomes. Standardization efforts, including the Image Biomarker Standardization Initiative (IBSI) [14], and the development of open-source tools such as PyRadiomics [15], have strengthened methodological rigor across studies. Prior CRC investigations have evaluated radiomics for tumor grading, nodal metastasis prediction, hepatic metastasis characterization, and correlations with molecular markers such as BRAF and KRAS [16,17,18]. However, many of these studies focused on isolated components of staging or relied on multi-sequence imaging not routinely acquired in clinical practice.

A typical radiomics workflow includes image acquisition and preprocessing, tumor segmentation, feature extraction, and machine learning-based modeling, followed by internal or external validation [8,14]. Major challenges include variability in acquisition parameters, lack of harmonization across scanners, and the high dimensionality of radiomic features—issues mitigated by normalization strategies, voxel size correction [19], feature reduction, and class-balancing techniques such as SMOTE [20]. Appropriate algorithm selection is equally important; ensemble methods such as Random Forests [21] and Gradient Boosting frequently yield robust performance in high-dimensional settings.

In this study, we investigated whether CT-based radiomics combined with machine learning can noninvasively predict both T and N categories in CRC. By systematically comparing preprocessing strategies, feature groups, and algorithms, this work aimed to identify robust radiomic signatures capable of refining preoperative staging and informing personalized treatment planning.

## 2. Materials and Methods

### 2.1. Patients

The study was approved by the Institutional Ethics Committee (CAAE number 65822222.4.0000.0030), and all patients provided informed consent. A total of 58 patients with colorectal adenocarcinoma (CRC) who underwent preoperative contrast-enhanced CT at our institution between 2022 and 2025 were initially screened.

The inclusion criteria were: (1) availability of preoperative contrast-enhanced CT images; (2) postoperative pathological confirmation of adenocarcinoma with TNM staging; and (3) no history of preoperative radiotherapy or chemotherapy.

Five patients were excluded due to incomplete imaging or clinical data, leaving 53 patients with CRC and a total of 55 tumors (two patients presented with synchronous tumors) for final analysis. The mean age was 62.6 ± 9.9 years (range, 40–87 years).

Baseline clinicopathologic data, including sex, age, preoperative carcinoembryonic antigen (CEA) level (normal ≤ 5 ng/mL; abnormal > 5 ng/mL), postoperative histologic grade, pathological TNM stage, and CT-reported tumor location (right-sided colon, left-sided colon, and rectum), were retrieved from institutional archives and are summarized in Table 1.

### 2.2. Assessment of TNM Status

Histopathological examination of all resected specimens was performed by the institutional pathology department. All cases were confirmed as colorectal adenocarcinoma (CRC). Pathologists were blinded to CT findings, and pathological TNM staging was determined postoperatively according to the American Joint Committee on Cancer (AJCC), 8th edition criteria [5]. The final classification was reported in the official pathology records.

### 2.3. Acquisition of CT Images

All patients underwent contrast-enhanced abdominal CT in our institution, covering the entire tumor of the colon or rectum. CT examinations were performed using either a 64-slice multidetector scanner (Aquilion 64, Toshiba Medical Systems, Ōtawara, Tochigi, Japan) or a 16-slice multidetector scanner (MX EVO2, Philips Healthcare, Amsterdam, The Netherlands). For dynamic contrast-enhanced CT, iodinated contrast medium was administered intravenously using a power injector. The arterial phase was acquired 20 s after contrast injection, followed by the portal venous phase at 60 s.

### 2.4. Radiomics Feature Extraction

Portal venous phase CT images were retrieved from the institutional picture archiving and communication system (PACS), as this phase provides superior delineation of tumor margins compared with adjacent normal bowel. CT scans were transferred from PACS to a local workstation for image analysis.

**Figure 1:**
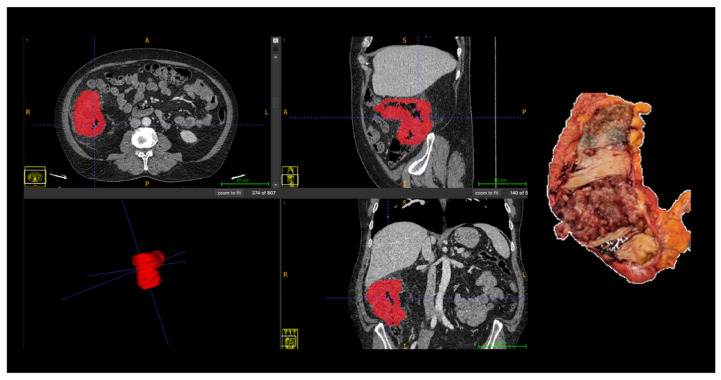
Manual segmentation of colorectal tumor (Patient 18) on portal venous phase CT and corresponding surgical specimen. The region of interest (ROI) was manually delineated (in red) on three consecutive slices using ITK-SNAP software, excluding intraluminal air, necrosis, vessels, and pericolic fat. Abbreviations: ROI, region of interest; CT, computed tomography; A, anterior; P, posterior; R, right; L, left, S, superior; I, inferior.

All segmentations were performed following a standardized institutional protocol by a single radiologist, using multiplanar visualization in ITK-SNAP software (version 3.8; www.itksnap.org) to ensure anatomical consistency. In cases with ambiguous tumor margins or complex anatomy, segmentations were supervised and reviewed by a senior abdominal radiologist to ensure anatomical accuracy. The region of interest (ROI) was delineated along the tumor boundary on three consecutive axial slices that best represented the lesion. Areas of intraluminal air, necrosis, vascular structures, and pericolic fat were carefully excluded from the contours (Figure 1).

The ROIs from the three largest consecutive cross-sectional images were integrated into a volume of interest (VOI). For each patient, the set of CT images (saved as multiple DICOM files) was exported into a single NRRD file using 3D Slicer (version 5.6.2; https://www.slicer.org). Three-dimensional radiomics feature extraction was performed using PyRadiomics (version 3.1.0; https://pyradiomics.readthedocs.io, accessed on 24 October 2024). A total of 107 quantitative features were extracted, including first-order statistics (18 features), three-dimensional shape-based features (14 features), and texture features derived from gray-level matrices, namely GLCM (24 features), GLRLM (16 features), GLSZM (16 features), NGTDM (5 features), and GLDM (14 features). For detailed information on the definition and calculation of individual radiomic features, please refer to the PyRadiomics documentation [22].

Prior to dimensionality reduction and model training, all radiomic features were standardized using z-score normalization, calculated as z = (x − μ)/σ, where x represents the original feature value, μ the mean, and σ the standard deviation of the corresponding feature. To prevent data leakage, the mean and standard deviation were computed exclusively from the training data within each iteration of the validation strategy and subsequently applied to the corresponding validation data.

### 2.5. Statistical Analysis

Statistical analysis and machine learning experiments were performed using R software (version 2024.12.1 + 563; http://www.R-project.org) and Python 3.7.12 with the scikit-learn library (version 1.5.1; https://scikit-learn.org). The scikit-learn framework was also used to estimate 95% confidence intervals (CI) for performance metrics.

Given the relatively small and imbalanced dataset, model performance was evaluated using a repeated hold-out validation strategy to reduce variability associated with a single data split. Specifically, tumors were randomly divided into training (75%) and validation (25%) sets, and this procedure was repeated for 100 iterations.

Model performance was evaluated using accuracy, sensitivity (recall), specificity, precision, F1-score, balanced accuracy, and area under the receiver operating characteristic curve (AUC), calculated using the True Positive (TP), True Negative (TN), False Positive (FP) and False Negative (FN) results. Accuracy was defined as (TP + TN)/(TP + TN + FP + FN). Sensitivity was calculated as TP/(TP + FN), specificity as TN/(TN + FP), and precision as TP/(TP + FP). The F1-score was defined as 2 × (Precision × Sensitivity)/(Precision + Sensitivity). Balanced accuracy was calculated as the mean of sensitivity and specificity, i.e., (Sensitivity + Specificity)/2. AUC was computed from the ROC curve obtained by plotting sensitivity against the false-positive rate.

To address class imbalance, the Synthetic Minority Over-sampling Technique (SMOTE) was applied exclusively to the training sets within each iteration. Feature standardization was performed using z-score normalization. Dimensionality reduction was conducted using principal component analysis (PCA) prior to model training to mitigate collinearity and reduce the feature-to-sample ratio.

Multiple supervised classification algorithms were evaluated within the same analytical framework, including logistic regression, k-nearest neighbors, naive Bayes, decision tree-based methods, ensemble classifiers, and linear discriminant analysis. This comparative approach was adopted to assess the robustness of different modeling strategies rather than to select a single optimized model.

### 2.6. Radiomics Signature Construction

**Figure 2:**
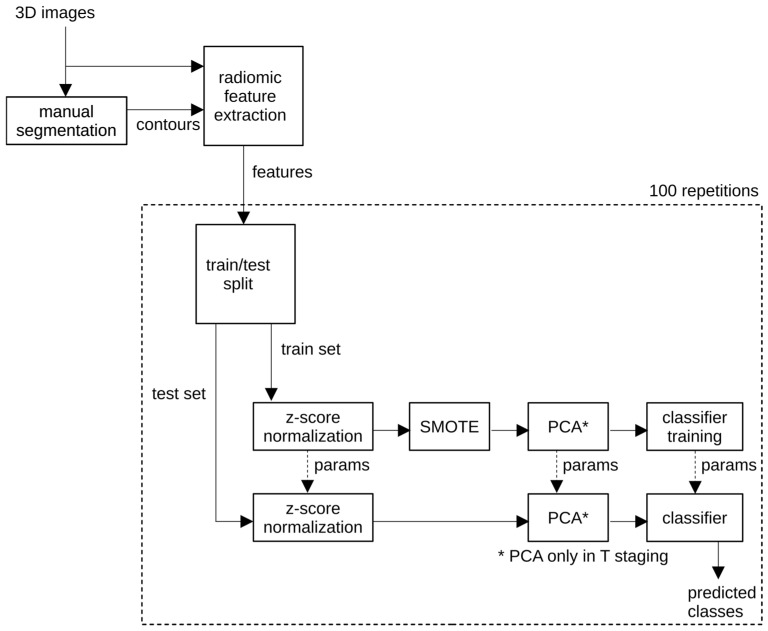
Workflow of the proposed radiomics classification pipeline.

Radiomics signatures were constructed using supervised machine learning pipelines, as summarized in Figure 2. Based on consistent performance across repeated validation experiments, one pipeline was selected as the proposed model for each classification task.

Machine learning experiments were performed using scikit-learn (version 1.5.1; https://scikit-learn.org). All models were trained to perform binary classification for tumor stage (T1–T2 vs. T3–T4) and nodal status (N0 vs. N+).

Multiple supervised machine learning classifiers were evaluated, including logistic regression (LR), k-nearest neighbors (KNN), Gaussian naive Bayes (NB), decision tree (DT), extra trees (ET), random forest (RF), multi-layer perceptron (MLP), AdaBoost (AB), gradient boosting (GB), linear discriminant analysis (LDA), and extreme gradient boosting (XGB). These models encompassed both linear classifiers and more complex ensemble-based approaches, enabling comparison between interpretable models and algorithms better suited for high-dimensional radiomics data.

All radiomic features were standardized using z-score normalization. To address class imbalance and improve classification stability, the Synthetic Minority Over-Sampling Technique (SMOTE) was applied exclusively to the training sets. Principal component analysis (PCA) was subsequently used for dimensionality reduction, minimizing feature redundancy while retaining the largest proportion of variance in the dataset.

In each iteration, the 55 segmented tumors were randomly split into a training set (75%) and a validation set (25%) using a class-wise stratified ShuffleSplit cross-validator to preserve class proportions. This procedure was repeated 100 times, and mean performance metrics along with 95% confidence intervals (CI) were reported. On average, each validation fold contained approximately 11–13 tumors for T-stage prediction and 13 tumors for N-stage prediction, depending on class distribution. This repeated resampling strategy was adopted to mitigate variability associated with small validation subsets while maintaining strict separation between training and validation data.

Based on the comparative evaluation of these classifiers, the proposed model for T-stage prediction (T1–T2 vs. T3–T4) consisted of a logistic regression classifier trained on shape-based radiomic features, with z-score normalization, dimensionality reduction using PCA, and class balancing using SMOTE. For N-stage prediction (N0 vs. N+), the proposed model consisted of an AdaBoost classifier trained on texture-based radiomic features, using z-score normalization and SMOTE, without dimensionality reduction, as PCA did not improve performance for this task.

### 2.7. Evaluation of Predictive Performance

Model performance was assessed using sensitivity (Se), specificity (Spe), balanced accuracy (BA), area under the ROC curve (AUC), precision (Pre), negative predictive value (NPV), F1-score, and accuracy (Acc). Validation was performed through repeated hold-out resampling with 100 iterations, and CIs were calculated using Student’s t distribution.

Experiments were conducted with various subsets of the extracted features (Table 2). These included experimenting with: each set independently; shape features combined with first order or texture features; combined set of texture features; and full feature set.

Comparative analyses were conducted across different preprocessing scenarios, in order to evaluate the incremental effect of each step on classification performance: (i) raw features, (ii) normalized features, (iii) normalized and PCA-reduced features, (iv) normalized features with SMOTE augmentation, (v) normalized and PCA-reduced features, followed by SMOTE augmentation, and (vi) normalized features with SMOTE augmentation followed by PCA reduction. The number of dimensions in the PCA-reduced subspace was 3, except in experiments with the combined set of texture features (texture75) and with the full feature set (fullset107), in which 5 dimensions were used instead.

### 2.8. Data and Code Availability

Radiomics feature matrices, preprocessing scripts, and machine learning pipelines used in this study were implemented in Python and R version 4.4.3. Due to ethical and privacy restrictions, raw CT images and identifiable clinical data cannot be publicly shared. However, derived datasets (radiomics features, model outputs, and preprocessing code) will be made available upon reasonable request to the corresponding author. No external datasets were deposited in public repositories; therefore, accession numbers do not apply.

### 2.9. Use of Generative Artificial Intelligence

Generative AI tools were used exclusively for language editing and organization. No AI tools were used for data analysis or scientific content creation.

## 3. Results

A total of 53 patients were included in the final analysis. The median age was 62 years (range, 40–87), and 31 patients (58%) were female. All patients underwent CT preoperative staging, and none received neoadjuvant chemoradiotherapy. Based on histopathological analysis, 11 patients (21%) were classified as T1–T2 (4 T1, 7 T2) and 42 patients (79%) as T3–T4 (28 T3, 14 T4). With regard to nodal staging, 20 patients (38%) were N0 and 33 (62%) were N+. No significant differences in age or sex distribution were observed between groups. These groups are detailed on Table 3.

**Figure 3:**
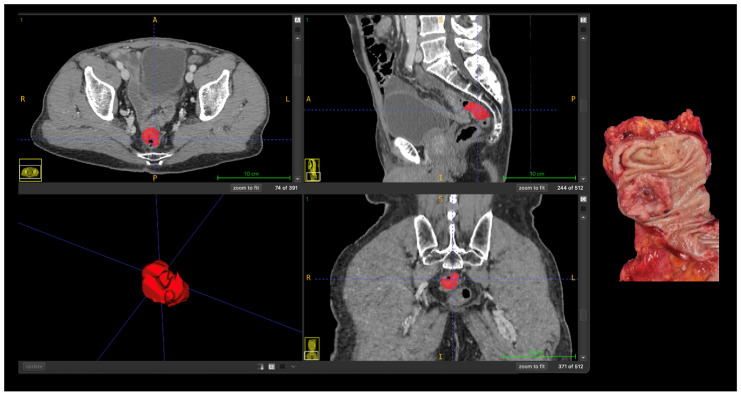
Correlation between radiomics-based tumor segmentation on contrast-enhanced CT and pathological anatomy in advanced (T4) colon cancer. Axial, coronal, and sagittal CT images with manual tumor segmentation (red); Three-dimensional rendering of the segmented tumor; Corresponding gross surgical specimen. Abbreviations: A, anterior; P, posterior; R, right; L, left, S, superior; I, inferior.

Representative case illustrating the correspondence between radiomics-based tumor segmentation on contrast-enhanced CT and the corresponding pathological anatomy are shown in Figure 3.

For T stage prediction (T1–T2 vs. T3–T4), a total of 990 experiments were conducted, spanning all combinations of 15 subsets of radiomic features, 6 preprocessing strategies, and 11 classifiers (15 × 6 × 11 = 990). In each experiment, 100 repetitions were performed, and the mean results were analyzed. Similarly, 990 experiments (with 100 repetitions) were conducted for N staging prediction (N0 vs. N+).

Due to class imbalance, the vast majority of the experiments resulted in high mean sensitivity; however, only a fraction of the experiments resulted in a reasonable balance of sensitivity and specificity. For simplicity, we labeled as “successful” those experiments which combined mean sensitivity greater than 0.70 and mean specificity greater than 0.50.

Table 4 presents an evaluation of the different preprocessing strategies. In T stage prediction, best results were achieved using normalized features with SMOTE augmentation followed by PCA reduction (26 out of 165 experiments were “successful”). In N stage prediction, best results were achieved using normalized features with SMOTE augmentation, without PCA reduction (37 out of 165 experiments were “successful”). PCA reduction was beneficial in T staging, but harmful in N staging. Due to severe class imbalance, SMOTE augmentation was pivotal in both scenarios.

Table 5 and Table 6 present the “success” rate of experiments conducted with the various subsets of radiomics features. In T stage prediction with preprocessing strategy (vi), best results were achieved using only shape-based features (8 out of 11 classifiers were “successful”); combining shape-based features with first order and/or texture features was not beneficial. In N stage prediction with preprocessing strategy (iv), best results were achieved using only texture-based features (for example, 7 out of 11 classifiers were “successful” using only GLCM features, and 6 out of 11 classifiers were “successful” using all texture features); combining texture-based features with shape-based features proved harmful.

**Figure 4:**
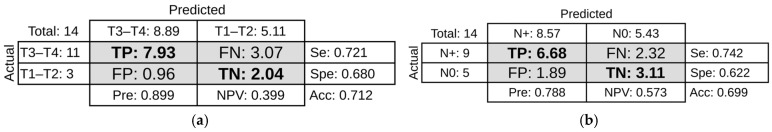
Confusion matrices showing model performance. (**a**) T stage prediction using logistic regression classifier with shape-based features and preprocessing strategy (vi). (**b**) N stage prediction using AdaBoost classifier with texture-based features and preprocessing strategy (iv). Each matrix displays the counts of true positive (TP), false positive (FP), true negative (TN), and false negative (FN) predictions. Performance metrics include sensitivity (Se), specificity (Spe), precision (Pre), and negative predictive value (NPV).

Table 7 shows the T stage prediction results achieved with each classifier, operating on the shape14 feature set with preprocessing strategy (vi). We highlight the result obtained with the logistic regression classifier: 0.721 mean sensitivity, 0.68 mean specificity, 0.70 mean balanced accuracy, and 0.751 mean AUC; the confusion matrix is shown in Figure 4a. 

Table 8 shows the N stage prediction results achieved with each classifier, operating on the texture75 feature set with preprocessing strategy (iv). We highlight the result obtained with the AdaBoost classifier: 0.742 mean sensitivity, 0.622 mean specificity, 0.682 mean balanced accuracy, and 0.75 mean AUC; the confusion matrix is shown in Figure 4b. 

## 4. Discussion

This study demonstrated that CT-based radiomics can provide clinically meaningful insights for the preoperative staging of colorectal cancer, particularly in differentiating T1–T2 from T3–T4 tumors. After applying normalization, PCA, and SMOTE on shape-based features, the best performance was achieved with the LR classifier, which provided a mean sensitivity of 0.721, specificity of 0.680, balanced accuracy of 0.70, and AUC of 0.751. Other classifiers, including KNN (AUC = 0.708) and LDA (AUC = 0.732), showed intermediate performance, while DT (AUC = 0.662) and XGB (AUC = 0.62) achieved lower discriminative ability. Importantly, ensemble models such as the ET classifier achieved the highest sensitivity (0.86) but at the expense of reduced specificity (0.473). These findings highlight the ability of CT-derived radiomic features, particularly shape-based descriptors, to capture morphological signatures of tumor invasiveness. By quantifying macroscopic attributes such as irregularity, elongation, and surface complexity, these features may indirectly mirror the microscopic patterns of wall infiltration and contour disruption that pathologists evaluate to define T categories. This correspondence suggests that shape-based radiomics could serve as an imaging correlate of histopathologic invasiveness, reinforcing their potential as noninvasive biomarkers for preoperative staging and treatment planning in colorectal cancer.

Prediction of nodal involvement (N0 vs. N+) achieved comparable performance to T staging. After applying normalization and SMOTE on texture-based features, the best performance was provided by the AB classifier, reaching a mean sensitivity of 0.742, specificity of 0.622, balanced accuracy of 0.682, and AUC of 0.75. Ensemble classifiers such as ET (AUC = 0.723) and RF (AUC = 0.721) also yielded robust outcomes, reinforcing the reproducibility of radiomic signatures across tasks. Notably, specificity increased slightly compared with the T-stage analysis, indicating a better harmony between true-positive and true-negative predictions. Consistent with this, the feature evaluation (Table 5) showed that texture-derived matrices such as GLCM, GLRLM, and GLSZM contributed most to nodal prediction, whereas shape features predominated in T-stage analysis. This pattern suggests that textural descriptors capture intratumoral heterogeneity—including variations in cellular density, necrosis, and stromal composition—that pathologists associate with biological aggressiveness and lymphatic dissemination. Accordingly, texture-based radiomics may serve as a quantitative imaging correlate of the tumor’s microenvironmental complexity, complementing shape features that primarily reflect local invasiveness. Nevertheless, as segmentation was limited to the primary tumor without direct nodal delineation, future models incorporating peritumoral or nodal regions may further enhance classification performance.

Prior studies reporting superior nodal-prediction performance, with AUCs ranging from 0.75 to 0.85, often employed direct or multiregional segmentation strategies. Liu et al. demonstrated that combining intratumoral and peritumoral MRI-based radiomics with clinical variables improved the prediction of lymph-node metastasis in rectal cancer, achieving an AUC of 0.83 in the validation cohort [23]. Similarly, Yuan et al. showed that a combined clinical–radiomic CT model integrating intratumoral and peritumoral features reached an AUC of 0.83 for nodal prediction in rectal carcinoma [24]. Eresen et al. also reported strong performance in colon-cancer nodal classification using direct lymph-node segmentation and an extreme gradient boosting model, achieving an AUC of 0.83, accuracy of 0.83, sensitivity of 0.81, and specificity of 0.85 [25]. Although our features were extracted exclusively from the primary tumor, our model achieved comparable discriminative performance, suggesting that intratumoral heterogeneity alone encodes meaningful biological information related to nodal spread.

Our findings are also consistent with other CT-based radiomics studies. Huang et al. developed and validated a CT-based radiomics nomogram integrating imaging features and clinical factors for preoperative lymph-node prediction in colorectal cancer, reporting C-indices of 0.736 in the training cohort and 0.778 in external validation [26]. Huang et al. demonstrated that a CT-based radiomics signature could differentiate between high-grade and low-grade colorectal adenocarcinomas, achieving an AUC of 0.812 in the training set, 0.735 in the validation set, and up to 0.895 in rectal-cancer subgroups [16]. Collectively, these studies support the robustness of CT-derived radiomics for primary-tumor characterization.

MRI-based radiomics has been more extensively explored in rectal cancer, often demonstrating superior performance for both T and N staging due to its higher soft-tissue contrast. Earlier MRI-based radiomics studies demonstrated that quantitative features derived from T2-weighted imaging could support preoperative T-stage prediction, with Ma et al. reporting an AUC of 0.82 using texture and shape-based radiomic features [17]. Subsequent work expanded this concept to nodal assessment, as histogram-based analyses on T2-weighted MRI achieved moderate predictive performance for lymph node metastasis, with reported AUC values ranging from 0.65 to 0.75, as demonstrated by Yang et al. [18].

More recently, radiomics research has shifted toward more refined modeling strategies. Ao et al. demonstrated that habitat-based multiparametric MRI radiomics can further improve discrimination between T2 and T3 rectal tumors by explicitly capturing intratumoral heterogeneity related to depth of invasion [27]. In parallel, Patanè et al. proposed updated MRI-based radiomics pipelines incorporating more robust validation strategies, confirming the reproducibility and clinical relevance of radiomics for rectal cancer staging in contemporary cohorts [28].

In comparison with these MRI-based approaches, our CT-based radiomics model achieved similar performance for T-stage prediction and comparable discriminative ability for nodal status, despite relying exclusively on tumor-only segmentation. These findings suggest that radiomic features extracted from routine contrast-enhanced CT can capture imaging biomarkers of both local invasion and lymphatic dissemination, supporting a complementary role for CT radiomics, particularly in clinical settings where MRI is unavailable or not routinely performed.

Clinically, accurate discrimination of T stage is crucial. Overstaging T2 tumors may expose patients to unnecessary chemoradiation and its associated morbidity, whereas understaging T3 lesions increases the likelihood of incomplete resection and recurrence. In routine clinical practice, CT is not considered a reliable modality for formal T staging in colorectal cancer, and radiologists do not routinely assign CT-based T categories, particularly in colon cancer. In our analysis, radiomics models achieved a mean AUC of 0.751, sensitivity of 0.721, and specificity of 0.68, indicating reliable performance in identifying advanced disease (T3–T4). This supports a potential complementary role for CT-based radiomics in scenarios where MRI availability is limited or impractical and CT remains the primary staging modality.

Nodal involvement prediction also achieved similar accuracy, with the AdaBoost classifier reaching an AUC of 0.750. Conventional CT-based nodal assessment is known to have limited accuracy and substantial interobserver variability, largely due to reliance on size-based criteria [29]. The performance observed in our study suggests that tumor-derived radiomic features encode biologically meaningful information related to lymphatic dissemination. Prior studies have demonstrated further improvements in nodal prediction by incorporating peritumoral or nodal radiomics, achieving incremental gains in AUC [23].

In right-sided colon cancer, accurate preoperative assessment of nodal status has direct surgical and prognostic implications. Unlike rectal cancer, where neoadjuvant strategies may alter the treatment sequence, surgery remains the first-line treatment for most colon tumors. Suspicion of nodal involvement may influence the extent of lymphadenectomy and the level of central vascular ligation, particularly in the context of complete mesocolic excision and minimally invasive surgical approaches [30]. In this setting, improved preoperative N staging may support surgical planning, risk stratification, and multidisciplinary decision-making. The ability of tumor-derived radiomic features to capture information related to lymphatic dissemination, as observed in our CT-based models, suggests a complementary role for radiomics in identifying patients at higher risk of nodal disease, especially when conventional size-based imaging criteria are limited [31].

Radiogenomic integration has also emerged as a promising direction. Lubner et al. showed that CT-derived texture features of colorectal liver metastases correlated with KRAS mutation status and clinical outcomes [32]. These radiogenomic strategies highlight the potential of imaging-derived features as noninvasive surrogates of tumor biology. Although promising, these approaches remain largely investigational in colorectal cancer and require validation in larger, prospective cohorts.

Methodologically, our pipeline adhered to established principles of radiomics research [14], incorporating standardized preprocessing (z-score normalization), dimensionality reduction to mitigate feature redundancy (PCA), and resampling strategies to address class imbalance (SMOTE). The reproducibility of results across classifiers and validation folds reinforces the robustness and stability of our approach. Accordingly, the evaluation of multiple feature sets, preprocessing strategies and classifiers should be interpreted as exploratory and hypothesis-generating rather than as confirmatory model optimization.

In future works, we will investigate which shape features are the most influential in T stage classification, and which texture features are the most influential in N stage classification. We will also work towards increasing sample size and reducing class imbalance. We expect that, by working with a reduced feature set, and a larger, more balanced, sample, our models will achieve higher sensitivity and specificity, resulting in better overall classification results across all metrics. We will also research novel and hybrid models from the literature and evaluate whether they can provide better results than the baseline model we used.

### 4.1. Potential Clinical Implementation of Model Outputs

Although this study is a proof-of-concept, the proposed classifiers can be readily deployed as a decision-support output by reporting, for each case, the predicted probability of advanced T stage (T3–T4) and/or node-positive disease (N+), together with the corresponding predicted class based on a prespecified threshold (e.g., 0.50). In practice, the report could provide statements such as: “Predicted probability of T3–T4 = p → predicted advanced T stage” and “Predicted probability of N+ = p → predicted node-positive status”, interpreted in light of the mean sensitivity/specificity observed in our internal validation. Future work with larger cohorts and external validation will be required to define clinically appropriate thresholds and to standardize how these probability outputs are presented for multidisciplinary decision-making.

### 4.2. Limitations

Nonetheless, several limitations of this study should be acknowledged. First, this was a single-center retrospective analysis with a relatively small and imbalanced cohort, particularly for early T-stage tumors, which may limit the generalizability of our findings. As this investigation was designed as a proof-of-concept study, no formal A Priori statistical power calculation was performed, and the limited number of early-stage cases reflects real-world surgical practice rather than selection bias.

Second, tumor segmentation was performed manually by a single radiologist, which may introduce observer dependency despite the use of standardized and widely validated software. Shape-based features are particularly susceptible to boundary variability and may therefore be more affected, whereas texture-based features derived from gray-level matrices (e.g., GLCM, GLRLM, GLSZM) are generally more robust to small contour variations. Future studies should include repeat segmentations and inter-reader agreement analysis using intraclass correlation coefficients, as well as semi-automated or automated segmentation methods, to further enhance reproducibility.

Third, the radiomics analysis was restricted to intratumoral features extracted from the primary tumor, as lymph nodes were not directly segmented, potentially limiting nodal-specific characterization.

In addition, although SMOTE was applied exclusively within the training sets to mitigate class imbalance, this technique does not increase the effective sample size of the validation data and should therefore be interpreted with caution. A repeated hold-out validation strategy was adopted to maximize data utilization while preserving strict independence between training and validation sets. While nested cross-validation represents a more rigorous framework—particularly for hyperparameter tuning in small datasets—it was not implemented in the present analysis, as most classifiers were trained using default parameters and dimensionality reduction via PCA was performed independently of outcome labels.

Although groupwise analyses consistently indicated that shape-based features were more informative for T-stage prediction and texture-based features for N-stage prediction, no formal feature-level selection or importance analysis was performed. As dimensionality reduction relied on PCA, individual feature contributions could not be directly interpreted, representing an additional limitation of the present study.

Finally, external validation across larger, multicenter cohorts and different imaging platforms remains essential before clinical translation. Future investigations incorporating nested cross-validation and external validation are warranted to further strengthen model robustness, generalizability, and clinical applicability.

## 5. Conclusions

In this study, CT-based radiomics demonstrated consistent and clinically meaningful performance in predicting both tumor (T) and nodal (N) stages of colorectal cancer. For T staging, the best-performing model—logistic regression—achieved a mean sensitivity of 0.721, a specificity of 0.68, a balanced accuracy of 0.70, and an AUC of 0.751, effectively distinguishing T1–T2 from T3–T4 tumors. This result was primarily driven by shape-based features, which quantitatively captured morphological patterns of wall invasion and contour irregularity, mirroring the criteria pathologists use to define T categories. Similarly, nodal prediction (N0 vs. N+) achieved comparable discriminative performance, with the AdaBoost model yielding a sensitivity of 0.742, a specificity of 0.622, a balanced accuracy of 0.682, and an AUC of 0.75. In this context, texture-based features emerged as the main contributors, reflecting intratumoral heterogeneity related to variations in cellular density, necrosis, and stromal composition—microscopic correlates of biological aggressiveness and lymphatic dissemination.

These findings underscore the potential of CT-based radiomics as a noninvasive adjunct to conventional staging, particularly in settings where MRI is not widely available or in colon cancer, where CT remains the primary modality. Future work should aim to integrate direct nodal and peritumoral segmentation, as well as multimodal fusion with MRI, molecular, and liquid biopsy biomarkers, to further enhance predictive performance and biological interpretability.

Overall, our results support CT radiomics as a promising framework for quantitative characterization of tumor invasiveness and dissemination, with potential applications in personalized treatment planning and risk stratification in colorectal cancer. Future investigations with larger, multicenter cohorts and nested cross-validation frameworks are warranted to confirm these findings and support clinical translation.

## Figures and Tables

**Table 1:** Baseline clinicopathologic characteristics of the study cohort (*n* = 53 patients, 55 tumors).

Characteristic	Value
Age, years (patients)	62.6 ± 9.9 (range 40–87)
Sex, *n* (%) (patients)	
Female	31 (58%)
Male	22 (42%)
Tumor location, *n* (%) (tumors)	
Right-sided colon	11 (20%)
Left-sided colon	27 (49%)
Rectum	17 (31%)
CEA level, *n* (% patients) *	
Normal (≤5 ng/mL)	29 (55%)
Abnormal (>5 ng/mL)	22 (41%)
Missing	2 (4%)
Histological grade, *n* (%) (tumors)	
Grade 1	17 (31%)
Grade 2	25 (45%)
Grade 3	5 (9%)
Missing	6 (11%)
Pathological stage, *n* (%) (tumors)	
T1	4 (7%)
T2	7 (13%)
T3	28 (51%)
T4	16 (29%)
N0	20 (36%)
N1	25 (45%)
N2	10 (18%)
M stage, *n* (%) (tumors)	
M0	55 (100%)
M1	0 (0%)

* CEA cutoff = 5 ng/mL; available for 53 patients.

**Table 2:** Subsets of radiomic features used in the experiments, showing the number of radiomic features per class in each subset. These are the total number of features computed by the PyRadiomics library in each class of features it implements.

Set\Class	First Order	Shape (3D)	GLCM	GLRLM	GLSZM	NGTDM	GLDM
firstorder18	18	-	-	-	-	-	-
shape14	-	14	-	-	-	-	-
glcm24	-	-	24	-	-	-	-
glrlm16	-	-	-	16	-	-	-
glszm16	-	-	-	-	16	-	-
ngtdm5	-	-	-	-	-	5	-
gldm14	-	-	-	-	-	-	14
shapefirstorder32	18	14	-	-	-	-	-
shapeglcm38	-	14	24	-	-	-	-
shapeglrlm30	-	14	-	16	-	-	-
shapeglszm30	-	14	-	-	16	-	-
shapengtdm19	-	14	-	-	-	5	-
shapegldm28	-	14	-	-	-	-	14
texture75	-	-	24	16	16	5	14
fullset107	18	14	24	16	16	5	14

**Table 3:** Baseline clinical characteristics of the study cohort (*n* = 53 patients).

Characteristic	Value
Age, years	62.6 ± 9.9 (range 40–87)
Sex, *n* (%)	
Female	31 (58%)
Male	22 (42%)
T stage, *n* (%)	
T1–T2	11 (21%)
T3–T4	42 (79%)
N stage, *n* (%)	
Negative (N0)	20 (38%)
Positive (N1–N2)	33 (62%)

**Table 4:** Evaluation of preprocessing scenarios for T and N stage prediction experiments. Numbers represent the percentage of “successful” experiments—i.e., those achieving mean sensitivity > 0.70 and mean specificity > 0.50—out of 165 experiments per task.

Preprocessing	T Staging	N Staging
raw	0.6	5
normalized	0.6	5
norm-PCA	1	4
norm-SMOTE	7	**22**
norm-PCA-SMOTE	10	10
norm-SMOTE-PCA	**16**	6

**Table 5:** Evaluation of individual classes of radiomic features for T and N stage prediction. Values represent the percentage of classifiers—out of 11—that achieved “successful” performance (mean sensitivity > 0.70 and mean specificity > 0.50).

Feature Set	T Staging *	N Staging **
firstorder18	0	9
shape14	**73**	0
glcm24	0	**64**
glrlm16	0	45
glszm16	0	45
ngtdm5	9	9
gldm14	9	27

* pre-processing: norm-SMOTE-PCA. ** pre-processing: norm-SMOTE.

**Table 6:** Evaluation of combined subsets of radiomic features. Values represent the percentage of classifiers—out of 11—that achieved “successful” performance (mean sensitivity > 0.70 and mean specificity > 0.50).

Feature Set	T Staging *	N Staging **
shapefirstorder32	18	0
shapeglcm38	9	18
shapeglrlm30	9	27
shapeglszm30	18	9
shapengtdm19	**73**	0
shapegldm28	18	18
texture75	0	**54**
fullset107	0	9

* pre-processing: norm-SMOTE-PCA. ** pre-processing: norm-SMOTE.

**Table 7:** T stage prediction results for different classification algorithms, using the shape14 feature set and preprocessing strategy (vi). Mean values and 95% confidence intervals are shown.

Classifier	Se	Spe	BA	AUC
LR	0.72 (0.69–0.75)	**0.68 (0.63–0.73)**	**0.70 (0.67–0.73)**	**0.75 (0.72–0.78)**
KNN	0.75 (0.72–0.78)	0.62 (0.57–0.66)	0.68 (0.66–0.71)	0.71 (0.68–0.74)
NB	0.70 (0.68–0.73)	0.59 (0.53–0.64)	0.65 (0.62–0.67)	0.70 (0.66–0.74)
DT	0.80 (0.77–0.83)	0.52 (0.47–0.58)	0.66 (0.63–0.69)	0.66 (0.63–0.69)
ET	**0.86 (0.84–0.88)**	0.47 (0.42–0.53)	0.67 (0.64–0.70)	0.70 (0.66–0.73)
RF	0.85 (0.83–0.88)	0.49 (0.44–0.55)	0.67 (0.64–0.70)	0.68 (0.65–0.72)
MLP	0.81 (0.78–0.84)	0.55 (0.50–0.61)	0.68 (0.65–0.71)	0.73 (0.69–0.76)
AB	0.77 (0.74–0.80)	0.51 (0.45–0.57)	0.64 (0.61–0.67)	0.69 (0.65–0.72)
GB	0.76 (0.73–0.79)	0.51 (0.45–0.56)	0.63 (0.60–0.66)	0.69 (0.65–0.72)
LDA	0.65 (0.62–0.68)	**0.68 (0.63–0.73)**	0.67 (0.64–0.70)	0.73 (0.70–0.77)
XGB	0.83 (0.80–0.85)	0.55 (0.50–0.60)	0.69 (0.66–0.72)	0.67 (0.64–0.71)

**Table 8:** N stage prediction results for different classification algorithms, using the texture75 feature set and preprocessing strategy (iv). Mean values and 95% confidence intervals are shown.

Classifier	Se	Spe	BA	AUC
LR	0.61 (0.58–0.65)	0.46 (0.41–0.50)	0.54 (0.51–0.56)	0.55 (0.52–0.58)
KNN	0.71 (0.67–0.74)	0.60 (0.55–0.64)	0.65 (0.63–0.68)	0.70 (0.68–0.73)
NB	0.46 (0.42–0.49)	**0.69 (0.64–0.73)**	0.57 (0.55–0.60)	0.62 (0.59–0.65)
DT	0.68 (0.65–0.71)	0.50 (0.45–0.55)	0.59 (0.56–0.62)	0.59 (0.56–0.62)
ET	**0.81 (0.78–0.83)**	0.52 (0.48–0.57)	0.66 (0.64–0.69)	0.72 (0.70–0.75)
RF	0.75 (0.72–0.78)	0.54 (0.50–0.58)	0.65 (0.62–0.67)	0.72 (0.69–0.75)
MLP	0.69 (0.66–0.72)	0.45 (0.40–0.50)	0.57 (0.54–0.59)	0.60 (0.57–0.63)
AB	0.74 (0.71–0.77)	0.62 (0.58–0.67)	**0.68 (0.66–0.71)**	**0.75 (0.72–0.78)**
GB	0.71 (0.68–0.74)	0.57 (0.52–0.61)	0.64 (0.61–0.67)	0.70 (0.67–0.73)
LDA	0.64 (0.61–0.68)	0.53 (0.49–0.57)	0.59 (0.56–0.61)	0.59 (0.56–0.63)
XGB	0.74 (0.71–0.77)	0.55 (0.50–0.59)	0.64 (0.62–0.67)	0.74 (0.71–0.77)

## Data Availability

The data presented in this study are available on reasonable request from the corresponding author due to privacy and ethical restrictions.

## Abbreviations

The following abbreviations are used in this manuscript:

AB	AdaBoost
Acc	Accuracy
AJCC	American Joint Committee on Cancer
AUC	Area Under the Receiver Operating Characteristic Curve
BA	Balanced Accuracy
CEA	Carcinoembryonic Antigen
CI	Confidence Interval
CRC	Colorectal Cancer
CT	Computed Tomography
DT	Decision Tree
ET	Extra Trees
FN	False Negative
FP	False Positive
GB	Gradient Boosting
GLCM	Gray Level Co-occurrence Matrix
GLDM	Gray Level Dependence Matrix
GLRLM	Gray Level Run Length Matrix
GLSZM	Gray Level Size Zone Matrix
IBSI	Image Biomarker Standardization Initiative
ITK-SNAP	Interactive Toolkit for Segmentation of 3D Medical Images
KNN	k-Nearest Neighbors
LDA	Linear Discriminant Analysis
LR	Logistic Regression
MLP	Multi-Layer Perceptron
NB	Naive Bayes
NGTDM	Neighboring Gray Tone Difference Matrix
NPV	Negative Predictive Value
PACS	Picture Archiving and Communication System
PCA	Principal Component Analysis
Pre	Precision
RF	Random Forest
ROC	Receiver Operating Characteristic
ROI	Region of Interest
Se	Sensitivity
SMOTE	Synthetic Minority Over-Sampling Technique
Spe	Specificity
TN	True Negative
TNM	Tumor–Node–Metastasis
TP	True Positive
VOI	Volume of Interest
XGB	Extreme Gradient Boosting
